# How skin achieves mechano-resistance for land movement: the critical role of ER sensing

**DOI:** 10.1186/s13619-025-00270-w

**Published:** 2025-11-14

**Authors:** Weihong Fu, Hua Li, Wenxiu Ning

**Affiliations:** Yunnan Key Laboratory of Cell Metabolism and Diseases, Center for Life Sciences, School of Life Sciences, Yunnan University, Kunming, China

## Abstract

To adapt to gravitational forces during the transition to terrestrial life, animals evolved specialized paw skin to withstand their body weight and allow for locomotion. In a recent *Cell* article, Di et al. demonstrate SLURP1 as an endoplasmic reticulum (ER) membrane protein that protects palmoplantar keratinocytes from mechanical stress by preserving SERCA2b activity and inhibiting the pPERK-NRF2 signaling under mechanical pressure.

## Main text

The evolutionary transition from aquatic to terrestrial habitats imposed a critical requirement on animals to adapt to the mechanical pressures exerted by gravity. The paws or feet, in particular, serve as primary load-bearing structures that directly support the entire body weight. In contrast to non-load-bearing regions such as the dorsal skin, it remains largely unknown how the skin in these pressure-bearing areas mechanically adapts to maintain tissue homeostasis.

The epidermis forms a physical and water-impermeable barrier that protects the body from external stresses, including mechanical force. This barrier is sustained through continuous epidermal turnover, driven by the proliferation and differentiation of basal progenitor cells into spinous and granular layers, and ultimately into corneocytes. The proliferation and differentiation rates of epidermal basal progenitor cells are tightly regulated by both internal and external niche cues, with mechanical stress being a pivotal modulator. Epidermal stem cells are directly exposed to a variety of mechanical cues, including ECM (extracellular matrix) stiffness, localized stretching from suprabasal layers, and tissue compression or tension. Notably, the ECM can be sensed by integrin complexes, which transduce these mechanical signals into intracellular biochemical pathways to modulate epidermal stem cell proliferation (Samuel et al. 2011; Li et al. 2022). Additionally, enhanced contractility in suprabasal differentiated cells, achieved via overexpression of constitutively active ARHGEF11, triggers non-cell autonomous hyperproliferation of basal cells through YAP activation (Ning et al. 2021). Moreover, external skin stretching, such as the use of a subcutaneous inflatable ‘skin expander’, also induces hyperproliferation of epidermal stem cells (Aragona et al. 2020). Mechanosensitive channels such as Piezo1 also promote skin thickening by detecting tension in stretched skin (Xue et al. 2025). Despite these advances, a critical gap remains in understanding how tissue in high-pressure regions maintain homeostasis under sustained mechanical loads, such as those imposed by gravity.

A groundbreaking study by Di et al. (2025) published in *Cell* addresses this gap by identifying SLURP1, a member of the Ly-6/uPAR protein family, an endoplasmic reticulum (ER) membrane protein that enables epidermal keratinocytes to adapt to mechanical compression in gravity-loaded areas such as paw skin (Di et al. 2025). The authors found that SLURP1 emerged during the aquatic-to-terrestrial transition, coinciding with the evolution of weight-bearing skin regions. Notably, loss-of-function mutations in human *SLURP1* gene cause autosomal recessive palmoplantar epidermal hyperplasia and hyperkeratosis. The authors performed sequentially sciatic/femoral denervation, hindlimb suspension, and load restoration to *Slurp1*-KO mice. They found that epidermal thickness in *Slurp1*-KO mice decreased to wild-type levels when the paw was suspended, confirming that plantar hyperplasia in *Slurp1*-KO mice is driven by mechanical compression rather than intrinsic developmental defects. They further showed that the hyperplasia following *Slurp1* depletion is independent of classical mechanosensitive ion channels such as Trpv4 and Piezo1, using either genetic ablation or pharmacological inhibition with GsMTx4. Moreover, the mechanotransductive transcription factors YAP and TAZ remained unchanged in the *Slurp1*-KO paw epidermis. This is surprising given that mechanical induction of epidermal stem cell proliferation is often mediated through nuclear YAP activation (Xue et al. 2022; Grzelak et al. 2023; Ning et al. 2021; Jiang et al. 2021). The absence of involvement of these conventional mechanotransduction pathways suggests a distinct mechanism regulating epidermal stem cell proliferation and differentiation under high-force conditions such as gravity.

Mechanistically, Di et al. characterized SLURP1 as an ER membrane resident protein with its C-terminus oriented toward the ER lumen. A mutation that abolishes SLURP1 secretion rescued the phenotype in *Slurp1*-KO mice, confirming that the membrane-bound but not the secreted SLURP1 protects keratinocytes from mechanical pressure. Further investigation revealed that *Slurp1* depletion induces ER stress by inhibiting the PERK pathway. Using a custom air pressure system to simulate mechanical compression, the authors showed that compression promotes PERK and eIF2α phosphorylation, which is suppressed by SLURP1 overexpression. Proteomic analysis identified an interaction between SLURP1 and SERCA2b, an ER calcium pump. Mechanical compression led to cytosolic calcium accumulation, which could be reversed by SLURP1 overexpression or treatment with the SERCA2 activator CDN1163. Importantly, CDN1163 also rescued epidermal thickening in *Slurp1*-KO mice, directly linking SLURP1 function to SERCA2b-mediated calcium balance. *Slurp1* depletion increased the expression of NRF2, a PERK downstream effector, whose depletion similarly rescued the hyperproliferative phenotype of *Slurp1*-KO mice. Collectively, these findings led the authors to conclude that SLURP1, as an ER membrane protein, protects epidermal keratinocytes from gravity-like mechanical stress by maintaining SERCA2b function and ER/cytosolic calcium homeostasis, thereby suppressing PERK-NRF2 signaling and limiting oxidative stress responses.

The study by Di et al. represents a major advance in the fields of mechanotransduction and developmental biology. However, this work also raises several important unanswered questions. First, although SLURP1 is localized to granular cells, it remains unclear how its depletion in these cells transmits signals to basal cells to change their proliferation and differentiation. Second, beyond gravity, the paw skin is also subjected to frictional forces during locomotion. This friction forces likely act synergistically with gravitational compression to exacerbate skin hyperplasia in *Slurp1*-KO mice. Additionally, how non-SLURP1 expressing cell types in the paw skin (e.g. dermal fibroblasts and adipocytes) respond to mechanical compression also remains unknown. Furthermore, while ER bound SLURP1 is sufficient for mechano-resistance, the role of secreted SLURP1 in immunomodulation and its potential contribution to non-mechanical phenotypes in patients warrants further investigation. Mechanistically, how does SLURP1 promote SERCA2b function to maintain ER-cytosol calcium balance under mechanical pressure? Does compression induce conformational changes of SLURP1, SERCA2b or both? Intriguingly, SERCA2b dysfunction in humans is linked to Darier’s disease, which is phenotypically distinct from SLURP1-related palmoplantar keratoderma (Moschella et al. 2025). Notably, both CDN1163 treatment and NRF2 depletion only partially rescued the hyperplastic phenotype in *Slurp1*-KO mice compared to hind paw suspension, implying that additional, uncharacterized factors are involved in the mechano-resistance. What is the physiological significance of this mechanical regulatory process? This work reframes palmoplantar keratoderma (PPK)—typically viewed as an irreversible genetic disorder—as a dynamic, mechanically driven condition. Hence, a straightforward and potentially therapeutic approach to alleviate symptoms in PPK patients may involve mechanical offloading (e.g., targeted foot loading reduction) combined with SERCA2b-activating drugs. A deeper understanding of the mechanobiology of stem cells and their niche will be essential for developing improved therapies for PPK and similar skin disorders.

## Conclusions

In conclusion, the work by Di et al. (2025) reveals an unprecedented role of ER in sensing non-classical mechanical compression during terrestrial locomotion. This finding underscores that distinct physical forces exert their effects on the skin epidermis through unique mechanisms. Moving forward, it will be critical for basic science to identify the key molecules that integrate these non-classical mechanotransduction cues at the molecular and cellular levels and to elucidate their specific roles in maintaining tissue homeostasis and driving regeneration.

## Data Availability

Not applicable.
